# Individualised niches: an integrative conceptual framework across behaviour, ecology, and evolution

**DOI:** 10.1002/brv.70147

**Published:** 2026-02-18

**Authors:** Oliver Krüger, Jaime Anaya‐Rojas, Mitja Back, Barbara Caspers, Nayden Chakarov, Melanie Dammhahn, Alkistis Elliott‐Graves, Claudia Fricke, Jürgen Gadau, Joseph I. Hoffman, Marie I. Kaiser, Sylvia Kaiser, Peter Korsten, Ulrich Krohs, Joachim Kurtz, Roland Langrock, Caroline Müller, Robert Peuß, Klaus Reinhold, Helene Richter, Norbert Sachser, Holger Schielzeth, Tim Schmoll, Ralf Stanewsky, Tamas Szekely, Franz J. Weissing, Meike Wittmann, Shuqing Xu

**Affiliations:** ^1^ Department of Animal Behaviour Bielefeld University Morgenbreede 45 Bielefeld 33615 Germany; ^2^ Joint Institute for Individualisation in a Changing Environment (JICE), Bielefeld University and University of Münster PO Box 100131 Bielefeld 33501 Germany; ^3^ Animal Evolutionary Ecology Group, Institute for Evolution and Biodiversity, University of Münster Hüfferstrasse 1 Münster 48149 Germany; ^4^ Department for Psychological Diagnostics and Personality Psychology University of Münster Fliednerstraße 21 Münster 48149 Germany; ^5^ Department of Behavioural Ecology Bielefeld University Morgenbreede 45 Bielefeld 33615 Germany; ^6^ Department of Behavioural Biology University of Münster Badestraße 13 Münster 48149 Germany; ^7^ Department of Philosophy Bielefeld University PO Box 100131 Bielefeld 33501 Germany; ^8^ Department of Animal Ecology Institute of Zoology, University of Halle‐Wittenberg Hoher Weg 8 Halle (Saale) 06120 Germany; ^9^ Molecular Evolution and Sociobiology Group, Institute for Evolution and Biodiversity, University of Münster Hüfferstrasse 1 Münster 48149 Germany; ^10^ Department of Evolutionary Population Genetics, Faculty of Biology Bielefeld University Bielefeld 33501 Germany; ^11^ Center for Biotechnology (CeBiTec), Faculty of Biology Bielefeld University Bielefeld 33615 Germany; ^12^ British Antarctic Survey High Cross, Madingley Road, CB3 OET Cambridge UK; ^13^ Edward Llwyd Building, Penglais Campus, Aberystwyth University Aberystwyth SY23 3BF UK; ^14^ Department of Philosophy University of Münster Domplatz 23 Münster 48143 Germany; ^15^ Department of Statistics and Data Analysis Bielefeld University PO Box 100131 Bielefeld 33501 Germany; ^16^ Department of Chemical Ecology Bielefeld University PO Box 100131 Bielefeld 33501 Germany; ^17^ Department of Evolutionary Biology Bielefeld University Morgenbreede 45 Bielefeld 33615 Germany; ^18^ Population Ecology Group Institute of Ecology and Evolution, Friedrich Schiller University Dornburger Straße 159 Jena 07743 Germany; ^19^ Institute of Neuro‐ and Behavioural Biology, University of Münster Röntgenstraße 16 Münster 48149 Germany; ^20^ Milner Centre for Evolution, Department of Biology and Biochemistry University of Bath Bath BA2 7AY UK; ^21^ Groningen Institute for Evolutionary Life Sciences University of Groningen Nijenborgh 7 Groningen AG 9747 the Netherlands; ^22^ Department of Theoretical Ecology Bielefeld University PO Box 100131 Bielefeld 33501 Germany; ^23^ Department of Evolutionary Plant Sciences Institute of Organismic and Molecular Evolution, University of Mainz Hanns‐Dieter‐Hüsch‐Weg 15 Mainz 55128 Germany

**Keywords:** animal personality, fitness, individualisation, individualised niche, integrative concept, niche

## Abstract

Individuals differ. While seemingly trivial, this insight has nevertheless led to paradigm shifts, as three key fields of organismal biology have seen marked changes in key concepts over the past few decades. In animal behaviour, it has become increasingly recognised that behavioural differences among individuals can be stable over time and across contexts, giving rise to the concept of animal personalities. In ecology, attention has similarly shifted towards variation in the ecological niches occupied by species, populations and individuals, giving rise to the concept of niche specialisation or individual niche variation. In evolutionary biology, where individual variation has always been central, there is a growing awareness of the complex and dynamic ways in which individuals interact with the environment to produce unique phenotypes. Additionally, recent theoretical and empirical research suggests that fitness landscapes are not only complex, with multiple fitness peaks, but might even be more accurately described as constantly shifting ‘fitness seascapes’, where the fitness peak that an individual can reach – whether local or global – depends on its genotype and its interaction with the environment. Moreover, the previous distinction between ecological and evolutionary timescales is being replaced by a more integrative view that recognises that evolution can occur on ecological timeframes. These shifting perspectives over the past two decades underscore the need for a more integrated conceptual framework that transcends disciplines. While in behaviour, ecology and evolution, the concept of individualisation has contributed to major scientific progress, sufficient cross‐fertilisation is still lacking. Here, we propose a new conceptual unification: the individualised niche. By merging the niche concept with the fitness concept, new explanatory power for both ecological and evolutionary processes emerges.

## I. INTRODUCTION

The simple but profound realisation that individuals differ was already made by Aristotle almost 2,500 years ago and is an unrefuted part of our lives. However, this realisation, so uncontroversial that most biologists would accept it without question (Dall *et al*., 2012), has led to marked paradigm shifts in the fields of animal behaviour, ecology and evolution (Bolnick *et al*., 2003; Wolf & Weissing, 2012; Costa‐Pereira & Shaner, 2024). An integrated conceptual framework transcending disciplines is currently emerging (Dammhahn *et al*., 2018; Edelaar & Bolnick, 2019; Trappes *et al*., 2022), but is far from complete (Pochevielle, 2015; Sales, Hayward & Loyola, 2021). Our goal is to help to close this gap by first briefly outlining these field‐specific paradigm shifts toward a greater emphasis on individuals. We then introduce our central idea of combining these field‐specific paradigms into the new concept of the individualised niche as a much‐needed extension of the classic Hutchinsonian niche. Finally, we will briefly outline the key implications of the individualised niche concept.

### (1) Animal behaviour

Because behaviour can change far faster than many other traits, it represents a particularly plastic component of the phenotype. It is thus a powerful and rapid catalyst of an organism's responses to environmental changes and altered selection pressures (Réale *et al*., 2010; Sih *et al*., 2010). This crucial role of behaviour has been explicitly recognised (Forsman, 2015; Rubenstein & Alcock, 2018; Trillmich, Müller & Müller, 2018), with Sih *et al*. (2010, p. 934) succinctly summarising that ‘Behavior resides in the central core of this association’ (i.e. the interaction between an organism and its environment). Hence, the explanatory power of behavioural variation with regard to interactions with the environment over both ecological and evolutionary timescales is well established (Dingemanse *et al*., 2010; Wolf & Weissing, 2012). However, the environmental circumstances that influence the uptake, integration and feedback between environmental information and the phenotype have not been investigated sufficiently to understand individual variation in behaviour and interactions with the environment. These processes represent an important step of individualisation by which the developing organism can adjust to predictable aspects of the environment in which it will reproduce and thus realise its fitness (West‐Eberhard, 2003; Sultan, 2007).

**Fig. 1:**
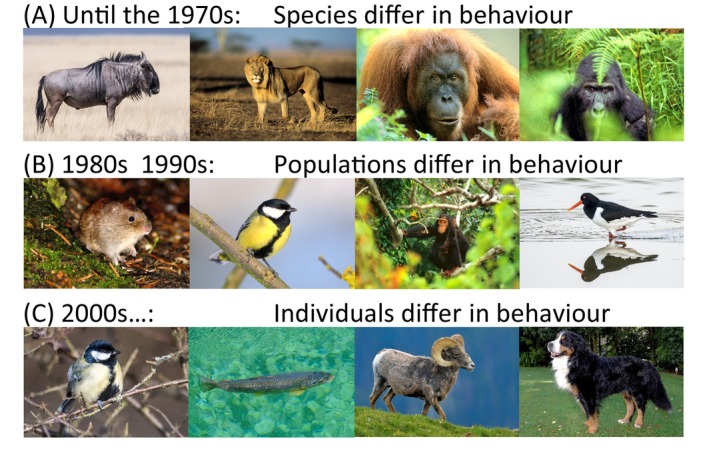
Illustrative timeline of the paradigm shifts in animal behaviour from (A) documenting behavioural differences between species until the 1970s as illustrated by key studies on ungulates (Jarman, 1974), carnivores (Schaller, 1972) and great apes (Galdikas, 1978; Fossey, 1983), followed by the recognition of (B) differences between populations of a given species in the 1980s and 1990s such as in house mice (*Mus musculus*, Butler, 1980), great tits (*Parus major*, Sherry & Galef, 1990), tool use differences in chimpanzees (*Pan troglodytes*, Boesch & Boesch, 1990) and foraging techniques in oystercatchers (*Haematopus ostralegus*, Goss‐Custard, 1996). The new century brought a further level of variation: (C) that between individuals of a given population as illustrated by studies on, for example, great tits (Carere *et al*., 2005), trout (*Salmo trutta*, Adriaenssens & Johnsson, 2013), bighorn sheep (*Ovis canadensis*, Poissant *et al*., 2013) and dogs (*Canis lupus familiaris*, Jones & Gosling, 2005). All photographs by Oliver Krüger.

The study of animal behaviour has undergone major shifts in the level of focus over recent decades. After Lorenz (1941) used behavioural differences between species to infer phylogenetic relationships, and when Tinbergen (1963) formulated his famous four questions, a species was understood to have a behavioural repertoire, with variation among individuals commonly being regarded as scatter around a species‐specific optimum (Wilson, 1998). This led to key monographs on a certain species' behaviour and comparisons with other species (Fig. 1A). With increasingly detailed behavioural studies emerging, scientists realised that, even within species, populations can differ markedly in their behaviour, whether this be foraging, tool use, or mating (Fig. 1B). This view was soon augmented with even more details, as many studies used either natural phenotypic variation or individual marking schemes to identify individuals and study their behaviour. Many of these individual‐based studies found considerable variation among individuals that was more than just random noise, or even detrimental deviation from a supposed fitness optimum (Fig. 1C, see also Freund *et al*., 2013; Childs *et al*., 2026).

Furthermore, behavioural plasticity was initially assumed to be almost limitless (Piersma & Drent, 2003), enabling organisms to respond appropriately to continuously changing environments [Sachser *et al*., 2020; see Eriksson *et al*. (2024) for a remarkable example of trophic plasticity in individual wolves, *Canis lupus*]. However, increasingly detailed individual‐based studies have revealed constraints to behavioural plasticity that have subsequently been well documented across diverse taxa from primates (including humans) to insects (e.g. Wilson, 1998; Sih *et al*., 2004; Williams, 2008; DeRango *et al*., 2019). These findings in turn gave rise to the concept of animal personalities (Sih *et al*., 2004; Réale *et al*., 2007). While animal personality can be considered to be a fundamental property of individuals (Freund *et al*., 2013), the more common notion in animal behaviour is that personality is a class of phenotypic variation that requires explanation and serves as a potent facilitator of further phenotypic differences (Wolf *et al*., 2007; Dochtermann & Dingemanse, 2013).

Since then, the focus has shifted towards understanding how genetic differences combined with differential life experiences can modify behavioural phenotypes, and towards understanding functional aspects of these differences in behavioural phenotypes. The widespread occurrence of animal personalities posed new explanatory challenges. While personality can constrain a trait and plasticity can maintain a degree of flexibility in the same trait, personality is commonly viewed as a constraint on behavioural plasticity and hence it was hard to explain how this could have evolved and why this could be selected for (Wolf *et al*., 2007; Wolf, van Doorn & Weissing, 2008; Wolf & Weissing, 2010; Dochtermann & Dingemanse, 2013). It has also become clear that variation in animal personality can be understood from an eco‐evo‐devo perspective (Stamps & Groothuis, 2010; Kappeler & Fichtel, 2015), which does not always mean that genetic differences are required (Freund *et al*., 2013). There is now considerable evidence that variation in personality can have a genetic basis and be subject to selection (Dochtermann, Schwab & Sih, 2015; Arroyo, Mougeot & Bretagnolle, 2017). This means that animal personality has profound implications for ecological and evolutionary processes (Wolf & Weissing, 2012). Not surprisingly, the call for a more integrative perspective has been heard. For example, the ‘pace‐of‐life syndrome’ concept (Ricklefs & Wikelski, 2002; Réale *et al*., 2010) explicitly links animal personalities to potential underlying endocrine mechanisms and recognises their ecological and evolutionary consequences (Niemelä *et al*., 2013; Dammhahn *et al*., 2018). The past decade has also seen efforts to merge animal personality with ideas from ecology and evolution (Araya‐Ajoy & Dingemanse, 2013; Sih *et al*., 2015; Dammhahn *et al*., 2018). Nevertheless, future studies and the development of a more extensive theoretical foundation are needed to explain the mechanisms underlying the co‐development of life‐history and personality traits (Mathot & Frankenhuis, 2018; Trillmich *et al*., 2018; Wright *et al*., 2019).

### (2) Ecology

In ecology, an increasing recent focus has likewise been on differences among individuals in a population. This shift from mean values of a population to individual diversity calls for a more flexible notion of the niche concept (Hutchinson, 1957) of a population or species (Bolnick *et al*., 2003; Holt, 2009; Violle *et al*., 2012; Layman, Newsome & Crawford, 2015; Costa‐Pereira *et al*., 2018). In addition, there has been a rather heated debate about the merits of Hutchinson's niche concept (Hubbell, 2001; Chase & Leibold, 2003), and Sales *et al*. (2021, p. 1) recently stated that ‘ecologists have long had a love‐hate relationship with the niche concept’.

What is nevertheless clearly emerging is that individuals specialise in and realise only subsets of the population's niche, and that these individual differences in niche realisation should reduce intraspecific competition. Conceptually, this is analogous to competition between species or populations (MacArthur & Levins, 1967). Today, there is strong evidence for niche specialisation or complementarity being a widespread phenomenon [e.g. invertebrates (Araújo & Gonzaga, 2007); fish (Evangelista *et al*., 2014; Skúlason *et al*., 2019); amphibians (Costa‐Pereira *et al*., 2018); reptiles (Figueroa *et al*., 2024); birds (Woo *et al*., 2008); mammals (Gharnit *et al*., 2020)]. Individuals can even change their niches during their lifetimes, giving rise to the idea of seasonal or ontogenetic niche shifts (Nakazawa, 2015; Carravieri *et al*., 2017; Figueroa *et al*., 2024).

With increasing empirical evidence, individual‐based ecology has not only been recently advocated (Grimm *et al*., 2025; Lu *et al*., 2025) but has also inspired a wave of conceptual and opinion papers over the past two decades highlighting when and where individual differences in niches might matter for ecological interactions among species (e.g. Bolnick *et al*., 2003, 2011; Araújo, Bolnick & Layman, 2011; Dall *et al*., 2012; Wolf & Weissing, 2012; Violle *et al*., 2012; Toscano *et al*., 2016; Moran, Wong & Thompson, 2017). This clearly suggests that individual variation matters (Bolnick *et al*., 2011; Des Roches *et al*., 2018). In contrast to the overt diversity of ‘ideas’ is the relative paucity of theoretical concepts and models [but see Lu *et al*. (2025) for a key recent modelling approach]. At present, we largely lack mechanistic models predicting the effects of intraspecific variation in traits, and particularly in labile traits such as behaviour, on ecological community structure and dynamics (although see Milles, Dammhahn & Grimm, 2020; Stump *et al*., 2022). Furthermore, the processes leading to niche specialisation are still not well understood. An additional perception leading to the conclusion that individuals matter greatly in ecology has come from ecosystem ecology in the notion of the keystone individual concept (Modlmeier *et al*., 2014). Directly transferred from the idea that some species have a disproportionate effect on a whole ecosystem, it proposes that some individuals have a disproportionate effect on entire groups or populations.

It has also become evident that individuals can actively shape or construct their ecological and social niches (Bergmüller & Taborsky, 2010) which in turn influences group and species composition and biodiversity (Hood & Larson, 2014). The important link between indirect genetic effects and social selection was established many years ago (Moore, Brodie III & Wolf, 1997) and can be a key factor in generating phenotypic diversity within a species, as the social environment modulates gene expression, physiology, behaviour, life history, and patterns of inheritance (Brooke *et al*., 2004; Schneider, Atallah & Levine, 2017). These effects can also be transgenerational and gave rise to the concept of social transgenerational plasticity (Hellmann & Sih, 2025) which can work without evoking parental effects. Thus, an individual can affect the phenotype of conspecifics, often leading to covariation between social and focal phenotypes. Hence, individuals are not distributed randomly with regard to their social niches, but they associate with each other based on their phenotypes. Such social niche construction may even drive co‐evolutionary episodes (Laland & Boogert, 2010; Schneider *et al*., 2017).

The reasoning outlined above has another crucial consequence. Whereas the ecological timescale was once deemed to be fundamentally different from the evolutionary one, this notion has now been replaced by a more integrative view where evolutionary change can indeed take place over ecological timescales (Hairston *et al*., 2005; Hanski, 2011; Hendry, 2016). Consequently, eco‐evolutionary feedbacks are now considered an important mechanism to facilitate rapid responses to changing conditions (Post & Palkovacs, 2009). Another integrative argument relevant to our idea is the concept of ecological opportunity (Wellborn & Langerhans, 2015). Here, the classic Hutchinsonian niche idea already integrates evolutionary processes. This concept links the niche to ecological opportunities, resulting in lineage formation, and diversifying selection can lead to adaptive divergence, phenotypic diversification and ultimately speciation. Over the last decades, we have therefore seen the merging of ecological and evolutionary concepts and evolutionary ecology has evolved into a powerful sub‐discipline.

What clearly emerges from these concepts is the need for further integration and additional empirical evidence to explore multiple aspects, such as the relationship between plasticity of behavioural and other phenotypic traits, including fitness measures, the ensuing diversity in ecological or social niches, and the resulting evolutionary scope for both adjustment and adaptation.

### (3) Evolution

Variation among individuals lies at the heart of Darwinian evolution, yet the causal basis of individual differences, whether or not they represent adaptations or noise with regard to the environmental conditions, and how individual differences impact on evolutionary processes, appears to be far more complex than previously assumed. Evolutionary biologists have found numerous examples where traits do not simply evolve towards one global fitness peak, but where many local fitness peaks exist (Gavrilets, 2004; Wagner, 2007, 2011; Oliveira, Taborsky & Brockmann, 2008; Williams, 2008). In addition, fitness peaks are not constant but subject to frequent change; hence fitness landscapes might be better described as fitness seascapes (Doebeli & Dieckmann, 2000; Mustonen & Lässig, 2009). This more dynamic view allows for diversification, as the context dependence of fitness allows for the emergence of polymorphism. Whether it is understanding the maintenance of polymorphism in a population (Krüger, Lindström & Amos, 2001) and/or how trait variation can be maintained by density‐dependent processes (van Benthem & Wittmann, 2020) or fluctuating selection (Calsbeek *et al*., 2013), it is clear that evolutionary fitness seascapes are frequency dependent. This implies that the optimal phenotype of an individual often depends on the phenotype distribution (Wagner, 2011). This is in no small part due to environmental heterogeneity, emphasising the role of ecology. As Grant & Grant (2008, p. 167), referring to Dobzhansky (1973), eloquently stated: ‘Nothing in evolutionary biology makes sense except in the light of ecology’. Post & Palkovacs (2009, p. 1629) elegantly phrased this crucial interaction as ‘interactions between the ecological theatre and the evolutionary play’. This has given rise to the research field of eco‐evolutionary feedbacks and dynamics (Pelletier, Garant & Hendry, 2009; Hendry, 2016, 2019).

As the trait values expressed by other individuals in a population can determine the optimal trait value of a given individual, they can influence that individual's fitness, giving rise to frequency‐dependent selection. We can only understand the evolution of traits and their variation if we incorporate both the particular ecological niche, but also the social niches that are realised by other individuals in the population. This goes beyond frequency‐dependent selection, as social niches are as dynamic as the behaviour of the individuals involved (Bergmüller & Taborsky, 2010). These social niches make the fitness seascape even more dynamic (Bergmüller & Taborsky, 2010; Saltz *et al*., 2016), thereby incorporating the adaptive value of different individual phenotypes collectively at the population level as well as offering a clear route for personality differences to evolve (Nicolaus *et al*., 2016). As argued by Edelaar & Bolnick (2019, p. 435), we need to ‘appreciate the multiple processes increasing individual or population fitness’.

## II. INDIVIDUALISED NICHES AS AN EXTENSION TO THE NICHE CONCEPT

The cornerstone ecological concept of the niche became more powerful when trait variation and the plasticity of individuals came into focus (Bolnick *et al*., 2003; Dingemanse & Wolf, 2013). Our new concept of an individualised niche builds on this foundation but is more dynamic and integrative, as it more explicitly recognises the two‐way interactions between individuals and their environment compared with phenotypic plasticity, and it also links the niche concept to the fitness concept. As an etho‐eco‐evo concept, it is applicable across behavioural, ecological and evolutionary timescales. Specifically, we define the individualised niche as: *a subset of the species' niche that arises from the interaction of the individual with its ecological and social environment*.

**Fig. 2:**
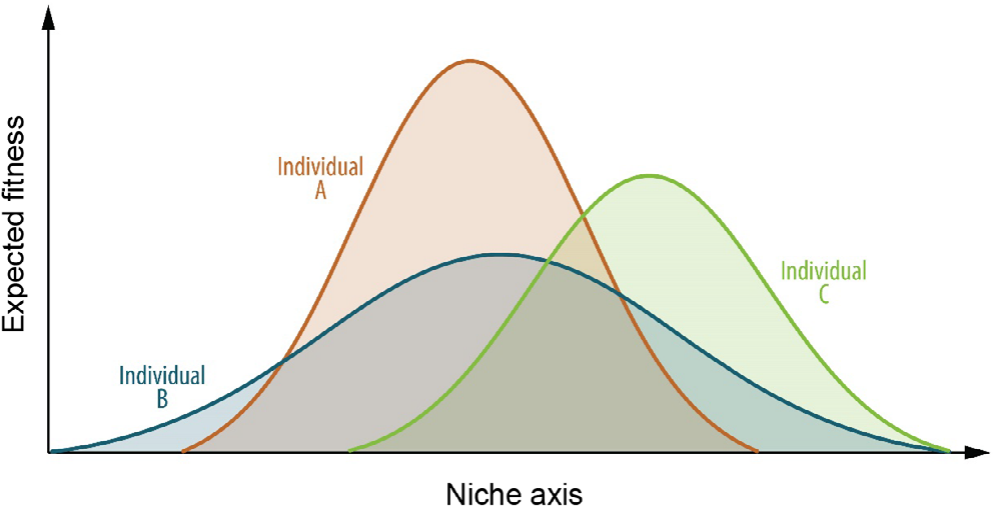
Illustration of the concept of individualised niches with three individuals depicted. Instead of plotting the likelihood of population persistence in relation to the values on a niche axis, as is commonly done when illustrating Hutchinsonian niches, we propose to use individual‐specific fitness expectations instead. Hence, the curves show how the fitness prospects of individuals differ in relation to the values on a niche axis. As fitness is difficult to measure in practice, fitness proxies like foraging success or expected lifetime reproductive success will often have to be used instead.

Like Grinnell's niche (Grinnell, 1917; Pocheville, 2015), the individualised niche links ecology to evolution and, like Hutchinson's niche (Hutchinson, 1957), it constitutes an n‐dimensional space that now describes how abiotic and biotic variables influence individual‐specific fitness functions. Interaction between an individual and its environment is key and has been recognised across vastly different disciplines (Kandler *et al*., 2024). Bergmüller & Taborsky (2010, p. 504) defined the social niche as ‘the social conditions an individual needs to practice its way of life’ and social niche specialisation has emerged as the term describing variation between individuals in their social niche (Montiglio, Ferrari & Reale, 2013). We broaden this definition to include all niche dimensions and propose the conceptual link to individual‐specific fitness (Fig. 2). We recognise that fitness is a complex concept (Metz, Nisbet & Geritz, 1992; Brommer, 2000) and that individual fitness expectations are difficult to estimate. In practical applications, we therefore advocate using fitness proxies, such as foraging success, life expectancy, fecundity, or lifetime reproductive success. As Hutchinson (1957) defined it, the (fundamental) ecological niche of a population is the range of environmental conditions in which a population can persist indefinitely. This implies non‐negative per capita population growth rate in the long term, hence the link between a Hutchinsonian niche concept and a fitness proxy is both reasonable and conceptually coherent.

The concept of an individualised niche, however, also allows for niche dimensions that the classic niche concept does not consider (Takola & Schielzeth, 2022). Take, for example, the idea of a social niche (Bergmüller & Taborsky, 2010; Montiglio *et al*., 2013; Saltz *et al*., 2016; Kaiser *et al*., 2024a). The social niche only makes sense if it is thought of as an individualised niche, because the social niche is different for each and every individual, depending strongly on individual experiences, personalities or individual differences in perception. As part of an ecological niche concept for the 21st century (Chase & Leibold, 2003; Holt, 2009), individualised niches should be considered (Müller *et al*., 2020; Trappes *et al*., 2022).

**Fig. 3:**
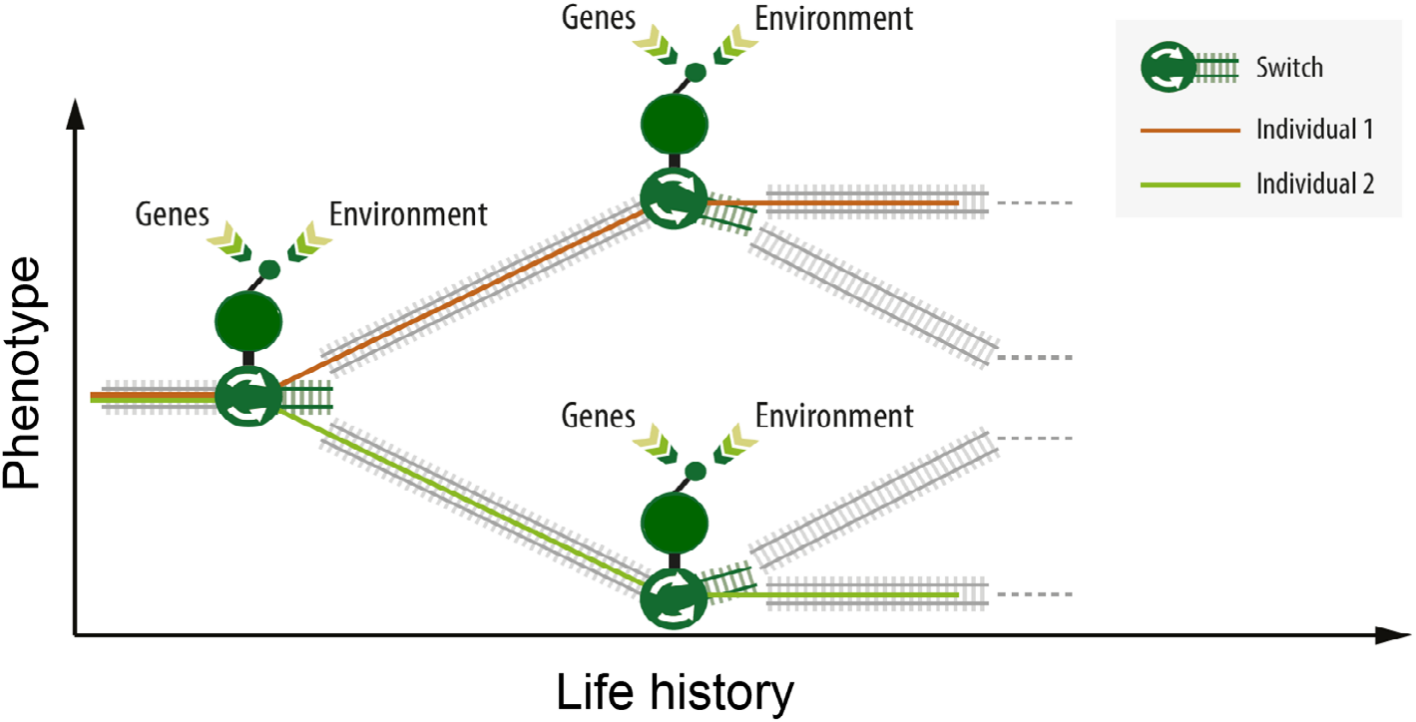
Scheme depicting the influence of genotype, environment and life‐history stage for the phenotypic expression of individual traits over the entire lifetime of two individuals. Phenotypic expression of a trait is significantly influenced by life‐history switch points where individuals change tracks according to the influence of both genes and the environment on the switch point.

How do we envision individualised niches emerging? The existing quantitative genetics framework to partition phenotypic variation into genetic, epigenetic and environmental components and their interactions provides the basis. There are important life events during early development and into adulthood where a shaping or re‐shaping of the phenotype can occur. Marked phenotypic changes are commonly observed at developmental switch points or phases in an animal's life (Sachser, Kaiser & Hennessy, 2013; Sachser *et al*., 2020). However, at the same time, at each switch point during the life history of an individual, phenotypic plasticity is constrained. Crucial constraints on phenotypic plasticity are, amongst others, the personality (Wolf *et al*., 2008; Dochtermann & Dingemanse, 2013) and neuroendocrine physiology (Taborsky *et al*., 2021) of an individual, but equally important constraints can be found in the ecological specialisation of individuals as well as in their evolutionary heritage. The picture that we envision is one of individuals ‘moving’ along tracks, encountering life‐history switch points that lead to changes of tracks (Fig. 3). These life‐history tracks constrain the phenotypic space of an individual, recognising that unconstrained phenotypic plasticity is unattainable. If life‐history switch points occur frequently, such as ontogenetic niche shifts (Nakazawa, 2015; Carravieri *et al*., 2017), these changes might even be viewed as almost continuous.

Three key processes give rise to individualised niches (Müller *et al*., 2020; Trappes *et al*., 2022): niche choice, niche conformance and niche construction (see also Odling‐Smee, Laland & Feldman, 2003; Stamps & Groothuis, 2010; Wade & Sultan, 2023 for similar terminology). We define these three key processes here: (*i*) niche choice is the process by which an individual chooses an environment that best matches its phenotype and thereby enhances its fitness; (*ii*) niche conformance is the process of phenotypic adjustment to the environment, resulting in a modified phenotype that better matches the environment; and (*iii*) niche construction is the process by which an individual makes changes to its environment, resulting in a modified environment that better matches its phenotype.

These three processes are distinct but not mutually exclusive. Niche choice results in a specific realised individualised niche. Alternatively, individuals can also conform to aspects of the environment, thereby changing their phenotype to achieve a better match to the environment and increase fitness. Again, this conformance results in a specific realised individualised niche. The phenotype thus becomes an individually tailored response to the environment that enhances an individual's fitness. Adaptive phenotypic plasticity, i.e. the ability of a genotype to produce different phenotypes, is an important mechanism of niche conformance. Finally, individuals can modify their environments in ways that benefit their fitness, thereby constructing their individualised niche.

These processes can influence an individual's position along the fitness function illustrated in Fig. 2. With regard to niche choice, the position of an individual along the niche dimension axis could either remain constant or shift due to choice. Regardless of the exact position along the niche dimension axis, niche choice should increase expected fitness, so that the realised individualised niche increases the individual‐specific fitness function. Niche choice can also contribute to the non‐random distribution of individuals along the niche dimension axis. With regard to niche conformance, an individual may be forced into a particular individualised niche and have to make the best of a ‘bad job’. Phenotypic plasticity is a key mechanism by which an individual can improve the match between the niche dimension and its phenotype, thereby increasing its expected fitness. Hence, the realised individualised niche increases the individual‐specific fitness function at that point. With regard to niche construction, individuals actively modify their environment and change the way in which the niche dimension relates to expected fitness. Hence, the shape of the individual‐specific fitness function changes. Niche construction can also contribute to the non‐random distribution of individuals along the niche axis.

**Fig. 4:**
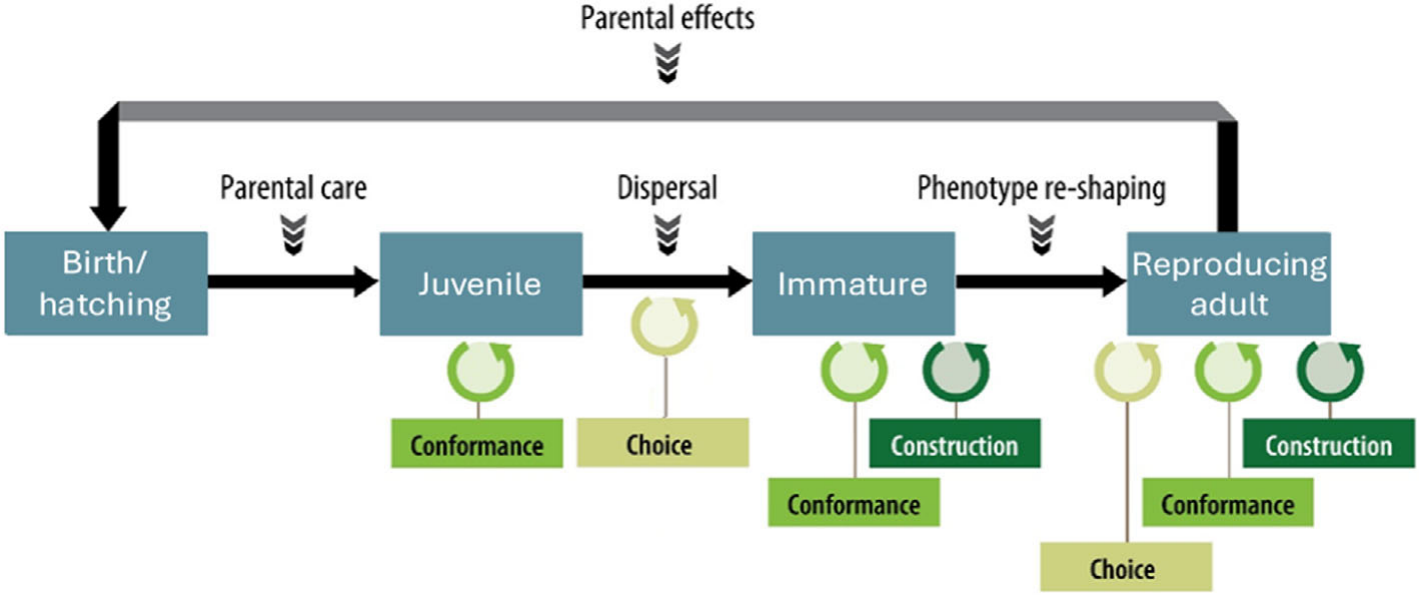
Idealised life cycle graph illustrating key switch points in life and where key processes leading to individualised niches act within the life cycle. Circular arrows depict when individuals commonly exercise niche choice, niche conformance, and niche construction which all affect the life history of an individual.

We envisage these three key processes typically operating to varying degrees at different stages of the life history of a typical (vertebrate) individual (Fig. 4): in many species, individuals receive some form of parental care after birth or hatching and they mostly (while acknowledging parent–offspring conflict) have to conform to the niche provided by the care‐giver(s). Typically thereafter, a moment follows when independence from parental care is achieved, which often coincides with dispersal, although effects of natal habitat and care are often still present (Davis & Stamps, 2004). At this stage, immature individuals can choose their individual niche, construct it, or, if necessary, they may have to make the best of a situation by conforming to it. In other species, no parental care is given but the parent determines where offspring will grow, enforcing even more niche conformance upon them, particularly in less‐mobile immature stages.

Between the immature and adult life stages, important re‐shaping of the phenotype can and does occur, often in so‐called ‘sensitive phases’ found across taxa (English & Barreaux, 2020; Sachser *et al*., 2020). Likewise, further choice, construction and conformance influences the phenotype of a fully mature, reproducing adult and its offspring *via* parental (i.e. inter‐ or trans‐generational) effects (Sánchez‐Tójar *et al*., 2020). All three processes are often mediated by info‐chemicals, such as hormones, pheromones, and allelochemicals (Müller *et al*., 2020), with both production and responsiveness changing throughout ontogeny. Their mediating power is hard to underestimate, yet remains little explored. More research is therefore needed to understand the (physiological) mechanisms underlying the processes of niche choice, conformance and construction.

Several other integrative approaches across behaviour, ecology and evolution have been increasingly advocated (Dall *et al*., 2012; Crusio, 2015; Gilbert, Bosch & Ledon‐Rettig, 2015; Kappeler & Fichtel, 2015; Skúlason *et al*., 2019). We believe there is considerable scope for the cross‐fertilisation of ideas, yet the need for new theoretical developments for the study of individualisation across these disciplines cannot be overlooked (Araújo *et al*., 2011; Bolnick *et al*., 2011; Matthews *et al*., 2014; Layman *et al*., 2015). The potential for integrating these concepts into a new theoretical framework has also been recognised (Poisot *et al*., 2011; Laland *et al*., 2014; Kappeler & Fichtel, 2015; Kuijper & Hoyle, 2015; Laubichler & Renn, 2015; Layman *et al*., 2015; Dammhahn *et al*., 2018; Edelaar & Bolnick, 2019), with entire journal issues having been devoted to topics such as ‘individual‐level niche specialization’ (*Oecologia* in 2015), ‘niche construction’ (*Evolutionary Ecology* in 2016) and ‘pace‐of‐life syndromes’ (*Behavioral Ecology and Sociobiology* in 2018).

While the literature now abounds with highly influential reviews (e.g. Bolnick *et al*., 2003; Réale *et al*., 2007, 2010; Wolf & Weissing, 2012; Layman *et al*., 2015; Edelaar & Bolnick, 2019), many fundamental questions remain unresolved, such as: (*i*) when during the lifespan and through which underlying physiological mechanisms do individual differences emerge (Wolf *et al*., 2007; Müller *et al*., 2020)? (*ii*) How are different cues integrated to produce individual phenotypes (Dall *et al*., 2005; Dall, McNamara & Leimar, 2015)? (*iii*) How are individual phenotypes shaped by experiences? (*iv*) How do different individual phenotypes coexist within a population (Wolf *et al*., 2007; Dingemanse & Wolf, 2010; Costa‐Pereira *et al*., 2018)? (*v*) What ecological and evolutionary consequences can be expected if individual trait variation and individualised niches are explicitly considered?

## III. IMPLICATIONS OF INDIVIDUALISED NICHES

Is there a need for yet another attempt to unify behaviour, ecology and evolution? What is to be gained by introducing the concept of individualised niches? The consequences of a more individualised view are manifold, some of which have already been described for animal personalities (Wolf & Weissing, 2012) and individual niche specialisation (Araújo *et al*., 2011; Lu *et al*., 2025). The concept of the individualised niche is another manifestation of the trend towards individualisation across organismal biology and even beyond (Kaiser *et al*., 2024b), but what new insights do we stand to gain in return? Five examples are described below.

Firstly, by using the individualised niche concept, a more refined understanding of species' interactions may be reached because individuals interact, not species. Individualised niches go beyond niche specialisation. While niche specialisation is an important extension of the cornerstone concept of the ecological niche, it largely remains within the domain of ecology. Recent papers (e.g. Carlson *et al*., 2021; Noss & Rosenblum, 2025) emphasise that both concepts focus on individuals and the ecological importance of studying individual variation. Even within the purely ecological perspective, the concept of individualised niches has a far stronger focus on individual trait variation and between‐individual variation than the niche specialisation concept. On top, this is combined with aspects of behaviour and evolution. A nice example of bridging the gap between ecology and evolution has recently been provided by Gubry‐Rangin *et al*. (2024). The individualised niche concept, however, goes further by bringing ecology and evolution into a single concept that makes clear predictions about the key processes at work, the life stages when they are most important, and suggests how to study individualisation empirically. While this shares some aims and content with Edelaar & Bolnick (2019), our framework is simple as it is built upon the cornerstone concept of the ecological niche, something that was very recently also strongly advocated by Lu *et al*. (2025). With individualised niches, one can better predict interactions through both facilitating traits, such as animal personality, and by integrating fitness proxies, allowing for a clearer understanding of the complex fitness seascape resulting from ecological and social environments (Schirmer *et al*., 2020).

**Fig. 5:**
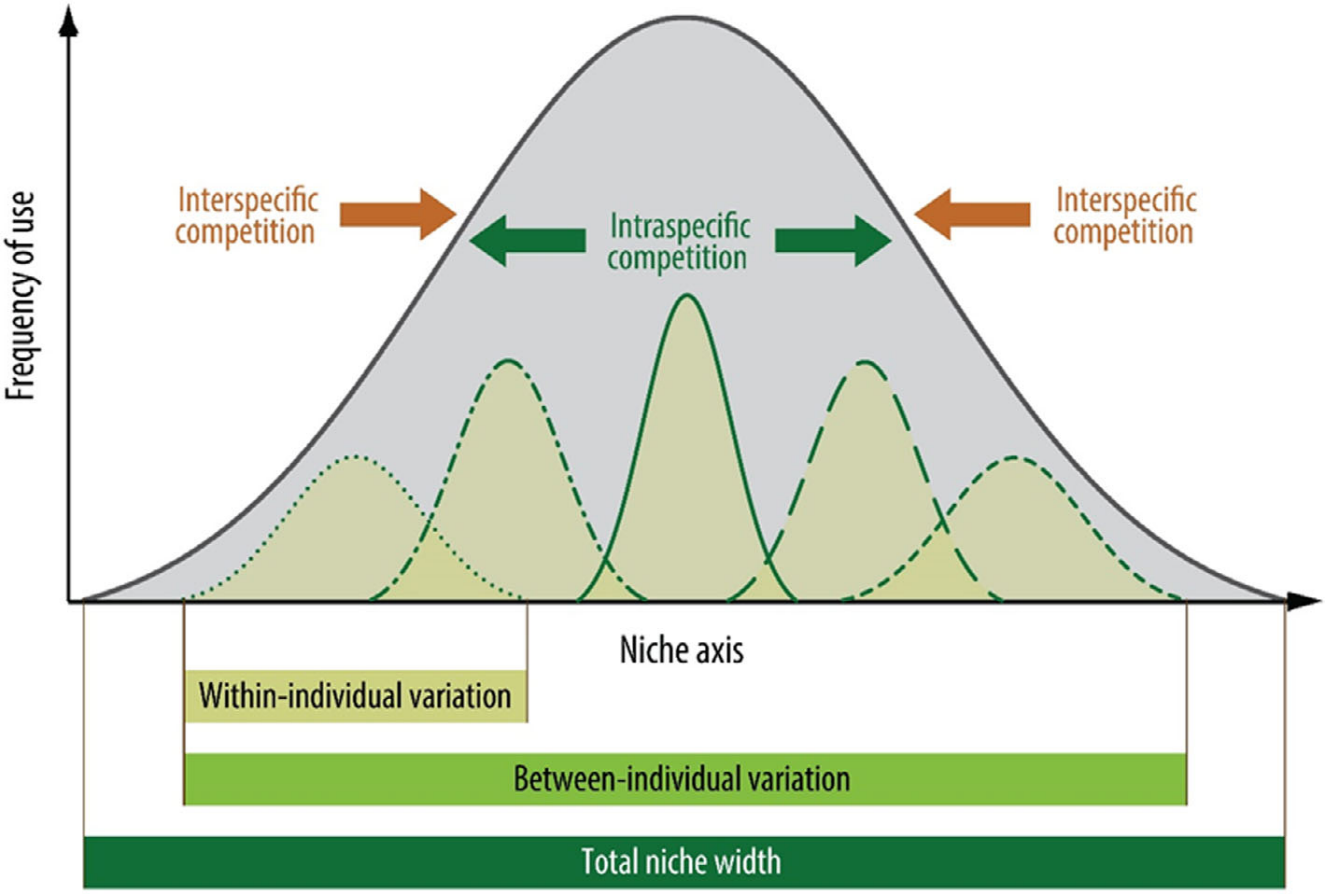
Illustration of the level of variation in niche space between and within individuals of a given population and how the two forces of intra‐ and interspecific competition either enlarge or compress the realised niche of a population. Modified after Bolnick *et al*. (2003).

Second, a more refined understanding of ecosystem structure and dynamics may be achieved when using the individualised niche concept because these dynamics emerge from individual interactions. Ecosystem ecology is already complex, yet to make it more predictive, individual variation needs to be taken into account (Violle *et al*., 2012). Evidence is accumulating that individual variation matters in community ecology (Bolnick *et al*., 2011; Barabas & D'Andrea, 2016; Hart, Schreiber & Levine, 2016; Kalirad & Sommer, 2024; Lichtenstein *et al*., 2024) and that individual niche specialisation arises from the complex interplay between intraspecific and interspecific competition (Fig. 5), which is particular to a given site (Costa‐Pereira *et al*., 2018). With the clear linkage of the individualised niche to fitness proxies, we gain predictive power, as we can better predict whether individual phenotypes and genotypes should persist in complex systems in the future. This perspective therefore paves the way for studying individual niche dynamics within an eco‐evolutionary framework.

Third, the individualised niche concept may help with individualised or precision conservation. Three aspects are particularly relevant here. First, individualisation might help populations to persist in the light of rapidly changing conditions. Analogous to conservation genetics, a viable population should encompass as many individualised niches as possible (Jeltsch *et al*., 2019; Schwarz *et al*., 2022). Second, in many cases we cannot conserve every individual in a given population (Bottrill *et al*., 2008). This makes it necessary to identify either keystone individuals, those most likely to benefit from conservation measures, or simply those individuals that are easiest to conserve given a finite budget. Finally, conservation measures can be tailored towards individuals, as in precision medicine, leading to ‘precision conservation’ (Desalle & Amato, 2017).

Fourth, individualised, or precision, animal welfare may benefit from the individualised niche concept. As for conservation, the trend towards individualisation might also bring about decisive new insights for the treatment of animals in human hands (Richter & Hintze, 2019). First, there is a general consensus that welfare is a characteristic of the individual animal (Broom, 2010). Indeed, individuals differ greatly in their social niches in captivity and hence in their proneness to develop welfare problems in general and in the types of problems they suffer from specifically (Koolhaas *et al*., 2010). Hence, it appears fundamental to investigate such inter‐individual differences systematically in order to understand why only some individuals develop welfare problems and others do not. Second, measures taken to improve animal welfare do not necessarily lead to the desired effect in all individuals of a group, as individuals differ both in their needs as well as in their acceptance of measures. Consequently, shifting the focus from a ‘one‐intervention‐fits‐all’ approach to individually tailored interventions should stimulate future developments in the field (Winckler, 2019).

Fifth, individualised genomics may both empower and mechanistically enhance our individualised niche concept. In parallel with developments in behavioural, ecological and evolutionary theory, new sequencing technologies now allow the screening of individuals of virtually any species for tens of thousands to millions of single nucleotide polymorphisms (Hu *et al*., 2021). Combined with recent advances in the theory and practice of genomic prediction (Daetwyler *et al*., 2013; McGaugh, Lorenz & Flagel, 2021; Ashraf *et al*., 2022; Howard, Jarquin & Crossa, 2022), we have now reached the point where it may be feasible to predict, or at least estimate, the phenotype of an individual from its genotype, even in wild populations where pedigrees are lacking. We acknowledge that our current understanding of gene–environment interactions is still relatively crude. However, predictive power may be improved by incorporating individual‐specific interactions with the environment in the form of individualised niches. Doing so could potentially yield higher estimates of heritability (*h* ^2^) and make predictions less noisy, thereby enhancing our understanding of individual variation and its role in microevolutionary processes and eco‐evolutionary dynamics.

## IV. CONCLUSIONS

(1) We would like to conclude with two quotes from key papers. Pocheville (2015, p. 547) wrote that: ‘The niche concept pervades ecology. Like the fitness concept in evolutionary biology, it is a core concept’. Lu *et al*. (2025, p. 1) wrote that: ‘The niche is a key concept that unifies ecology and evolutionary biology’. However, we can refine the second quote as we believe that the niche concept, in an individualised version as we and Lu *et al*. (2025) propose, *can further* unify ecology and evolutionary biology.
(2) By integrating individual differences in animal behaviour and considering the full developmental trajectory and its effects on an individual's phenotype, we propose that the individualised niche represents a novel, integrative concept for studying the causes and consequences of individualisation.
(3) Phenomena at the population or species level can be better understood by explicitly considering individual variation. The niche concept will undoubtedly continue to play a crucial role in ecology, but it needs to be updated (Chase & Leibold, 2003; Holt, 2009; Letten, Ke & Fukami, 2017; Sales *et al*., 2021; Lu *et al*., 2025).
(4) It is our belief that the etho‐eco‐evo concept of individualised niches can contribute toward this endeavour.

## Data Availability

The data that support the findings of this study are available on request from the corresponding author. The data are not publicly available due to privacy or ethical restrictions.
